# P K-Edge XANES Calculations of Mineral Standards: Exploring the Potential of Theoretical Methods in the Analysis of Phosphorus Speciation

**DOI:** 10.1021/acs.inorgchem.3c01346

**Published:** 2023-06-29

**Authors:** Alessandro Tofoni, Francesco Tavani, Ingmar Persson, Paola D’Angelo

**Affiliations:** † Department of Chemistry, Sapienza University of Rome, P.le A. Moro 5, 00185 Rome, Italy; ‡ Department of Molecular Sciences, Swedish University of Agricultural Sciences, P.O. Box 7015, SE-750 07 Uppsala, Sweden

## Abstract

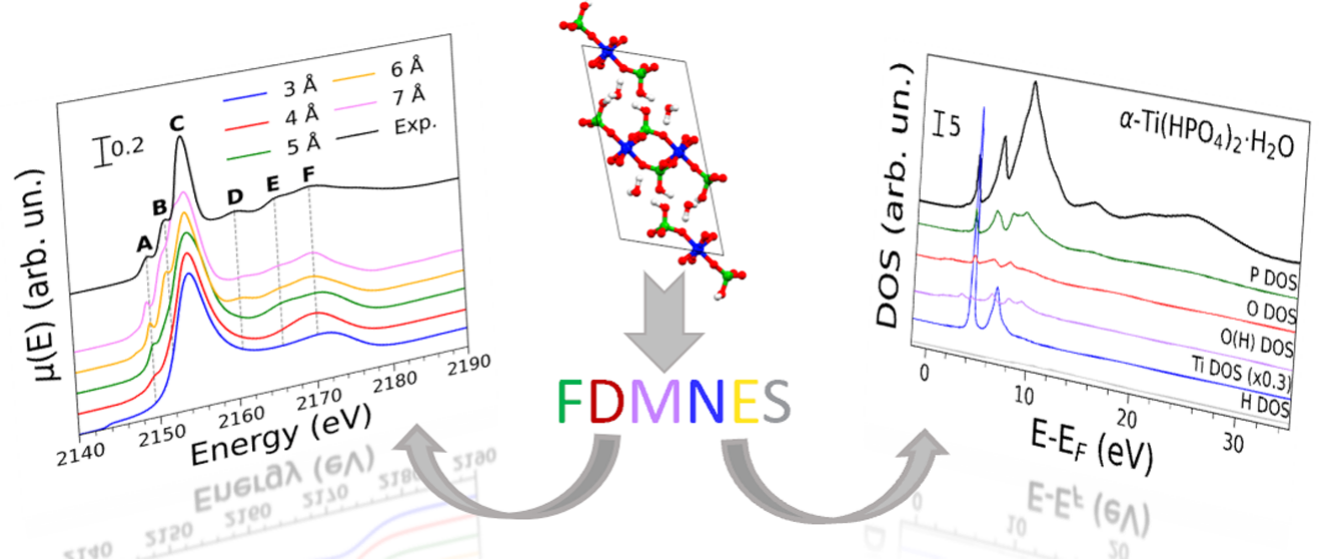

Phosphorus K-edge X-ray absorption near-edge structure (XANES) spectroscopy is a technique routinely employed in the qualitative and quantitative analysis of phosphorus speciation in many scientific fields. The data analysis is, however, often performed in a qualitative manner, relying on linear combination fitting protocols or simple comparisons between the experimental data and the spectra of standards, and little quantitative structural and electronic information is thus retrieved. Herein, we report a thorough theoretical investigation of P K-edge XANES spectra of NaH_2_PO_4_·H_2_O, AlPO_4_, α-Ti(HPO_4_)_2_·H_2_O, and FePO_4_·2H_2_O showing excellent agreement with the experimental data. We find that different coordination shells of phosphorus, up to a distance of 5–6 Å from the photoabsorber, contribute to distinct features in the XANES spectra. This high structural sensitivity enables P K-edge XANES spectroscopy to even distinguish between nearly isostructural crystal phases of the same compound. Additionally, we provide a rationalization of the pre-edge transitions observed in the spectra of α-Ti(HPO_4_)_2_·H_2_O and FePO_4_·2H_2_O through density of states calculations. These pre-edge transitions are found to be enabled by the covalent mixing of phosphorus s and p orbitals and titanium or iron d orbitals, which happens even though neither metal ion is directly bound to phosphorus in the two systems.

## Abstract 2

Calculating a P K-edge XANES spectrum with the FDMNES program starting from a crystallographic structure leads to excellent agreement with the experimental data and DOS calculations that enable accurate interpretation of the observed transitions.

## Introduction

Over the last two decades, P K-edge X-ray absorption near-edge structure (XANES) spectroscopy has become a routine tool for the analysis of phosphorus speciation in an increasing number of scientific fields. These include solution chemistry,^1^ catalysis,^2^ wastewater treatment,^3,4^ dairy manure or marine sediments analysis,^5,6^ evaluation of radiation damage on DNA,^7^ characterization of exhaust particulates,^8^ and investigations on the interaction between heavy metals and microorganisms.^9^ Medical applications such as bone analysis in osteoarthritic knees have also been reported.^10^ In this wide range of disciplines, P K-edge XANES has found its most popular and successful employment in environmental sciences, especially because of its ability to identify and quantify a variety of phosphorus species in a nondestructive manner.^11,12^ This technique has been, in particular, extensively used for the analysis of soils and to evaluate the performance of fertilizers.^13−17^ In these applications, protocols based on principal component analysis (PCA),^18−20^ as well as on linear combination fitting (LCF),^21−24^ have been employed for the data analysis. The latter procedure is in particular widely adopted, but it is also highly susceptible to errors and should be judiciously employed. Cases in which LCF has led to an incorrect quantification of phosphorus species have been reported in the literature,^25^ and this method is known to require extensive validation before it can be employed effectively.^26^ The fundamental issue of LCF-based approaches is that they rely on the assumption that a given compound yields the same spectrum when in pure form and when present as a component in a mixture. This is not necessarily true, as alterations of the crystal lattice leading to spectral distortions can occur in mixtures, especially in natural samples. Moreover, detecting and quantifying compounds that are not included in the LCF reference database is cumbersome.

At present, the analysis of P K-edge XANES data is mainly carried out on a qualitative basis, relying on the comparison between the X-ray spectra of reference compounds and those collected on the investigated samples, with little quantitative structural and electronic information being retrieved. These shortcomings could potentially be overcome by approaches relying on the quantitative theoretical analysis of P K-edge spectra, which only require a structural model of the examined compound to be carried out. However, although both the theoretical background and the necessary software resources for the rigorous calculation of XANES spectra are currently available,^27,28^ they have been seldom applied to the analysis of phosphorus compounds. As a matter of fact, an extremely limited amount of simulated P K-edge XANES spectra has been reported,^7,29,30^ and the agreement with the experimental data is not completely satisfactory in many cases.^31,32^ The majority of the reported quantitative P K-edge X-ray absorption studies instead focus on inspecting the extended X-ray absorption fine structure (EXAFS) region of the spectra,^33−35^ obtaining quantitative structural information with very high accuracy. Still, XANES analysis can provide important insights into the electronic structure of chemical systems, which can hardly be disclosed by EXAFS, due to the occurrence of peculiar transitions to bound states in the near-edge region of the spectra. Their shape depends on the electronic structure of the photoabsorber, namely its spin and oxidation states, as well as on the mixing of its valence orbitals with those of nearby species.^1,36^

The aim of this paper is to provide a step forward in the analysis of experimental P K-edge data, laying out a solid background for the calculation of P K-edge XANES spectra, while also revealing the structural and electronic contributions giving rise to the observed XANES features. Additionally, our results will contribute to the improvement of the currently available protocols for the determination of phosphorus speciation, which could benefit from rational applications of theoretical calculations. We consider a diverse set of phosphorus minerals, including some commonly employed as standards in the analysis of soils, namely, NaH_2_PO_4_·H_2_O, AlPO_4_, α-Ti(HPO_4_)_2_·H_2_O, and FePO_4_·2H_2_O. These exhibit marked differences in chemical composition as well as crystal structure, but not in phosphorus oxidation state (V) or local coordination environment that is tetrahedral in all cases. We will highlight the sensitivity of P K-edge XANES spectra to the intermediate-range structure of these compounds beyond the first coordination sphere of phosphorus, applying state-of-the-art theoretical XANES calculations to understand the structural contributions that shape the XANES signal. Further, we employ density of states calculations to shed light on the role of electronic contributions in phosphorus K-edge spectra, showing the role of covalent interactions in originating the pre-edge features often observed in P K-edge spectra of transition metal (TM) phosphates.

## Materials and Methods

Sodium dihydrogen phosphate monohydrate, NaH_2_PO_4_·H_2_O, was purchased from Merck at analytical grade quality and used without further purification. Titanium bis(hydrogenphosphate) dihydrate, α-Ti(HPO_4_)_2_·H_2_O, was synthesized in aqueous conditions according to a procedure described elsewhere.^4^

### X-ray Absorption Data

The P-edge XANES spectra analyzed in this study have been reported in already published papers,^1,4,26^ where the experimental conditions and procedures, and energy calibration, have been described in detail. All data were collected at the bending magnet beamline 8 at the Synchrotron Light Research Institute (SLRI) in Nakhon Ratchasima, Thailand.^37^

### Theoretical XANES Calculations and DOS Analysis

Advanced theoretical calculations of the XANES spectra have been carried out using the finite differences method near-edge structure (FDMNES) program starting from the crystallographic structures of the investigated systems available in the literature.^22,38−41^ FDMNES is a density functional theory (DFT) code that is able to calculate X-ray absorption spectra within both the full multiple scattering (MS) and finite differences method (FDM) theoretical frameworks.^42,43^ Its most recent versions implement efficient sparse solver algorithms parallelized under the MPI protocol,^28^ significantly lowering the computational cost of FDM calculations. The real Hedin–Lundqvist exchange-correlation potential has been used to take correlation effects into account,^44^ while the final-state broadening has been modeled by performing a convolution of the calculated spectra with a Lorentzian function of variable width Γ_tot_. Γ_tot_ is expressed as the sum of a tabulated core–hole lifetime width Γ_hole_ and an arctangent function Γ(ω)
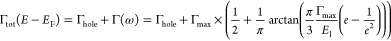
1where *E*_F_ is the Fermi energy; Γ; and Γ_max_, *E*_l_, and *E*_ctr_ are empirical parameters representing the final-state width, the width of the arctangent function, and its center, respectively. A fixed-width Gaussian broadening can be applied to account for the experimental resolution.

FDMNES is also able to compute the atom-projected and angular momentum resolved electronic density of states (DOS) δ(*E*) through integrals of the MS matrix elements τ_*l*,*m*_^*l*^′^,*m*^′^^
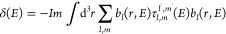
2where *b*_*l*_(*r*, *E*) represents the energy-dependent radial atomic wave function of the angular momentum *l*.^42^ DOS calculations can be useful to highlight the presence of isoenergetic states localized on different atoms, indicating orbital mixing.

For each structure, we performed a reconstruction of the crystallographic structure up to a certain cutoff distance from the photoabsorber with FDMNES. To exclude the presence of fragments, we included in the final clusters the full water molecules or (dihydrogen, monohydrogen)phosphate ions whose central O or P atoms, respectively, were located inside the given cutoff sphere. For each compound, a theoretical spectrum has then been calculated at the level of theory that could best reproduce the experimental data. Magnetic and dipole-allowed transitions have been included in the calculations. Each calculation was carried out as follows: the spectrum of NaH_2_PO_4_·H_2_O has been simulated using the structure reported in ref (38); for AlPO_4_, we used the structure determined by Muraoka and Kihara using the same settings as NaH_2_PO_4_·H_2_O.^40^ For FePO_4_·2H_2_O and FePO_4_, we employed the structures reported by Song et al. and Andersson et al., respectively, performing the calculations at the FDM level and applying a Gaussian broadening of 2.0 eV in order to account for the experimental resolution.^22,41^ For α-Ti(HPO_4_)_2_·H_2_O, the calculation was performed at the MS level, using the structure determined by Salvadó et al.^39^ Γ_max_ has been set to 1.5 eV, while *E*_l_ and *E*_ctr_ have been kept at their default value of 30 eV in all calculations. The atom- and angular momentum-projected DOS was also calculated for all systems.

We remark that DFT-based theoretical XANES spectra such as those computed by FDMNES exhibit an energy shift with respect to the experimental spectra both for the entire energy scale and the relative position of spectral features. This is known to be due to the omission of atomic and relativistic stabilization effects, as well as to systematic errors caused by the exchange-correlation potentials.^45,46^ This effect has been corrected for each calculated XAS spectrum reported in this work by aligning the energy of the theoretical white line transition to that of the experimental one. The applied energy shifts (which are all below or equal to 2.0 eV) are reported in Table S1.

## Results and Discussion

### NaH_2_PO_4_·H_2_O System

**Figure 1:**
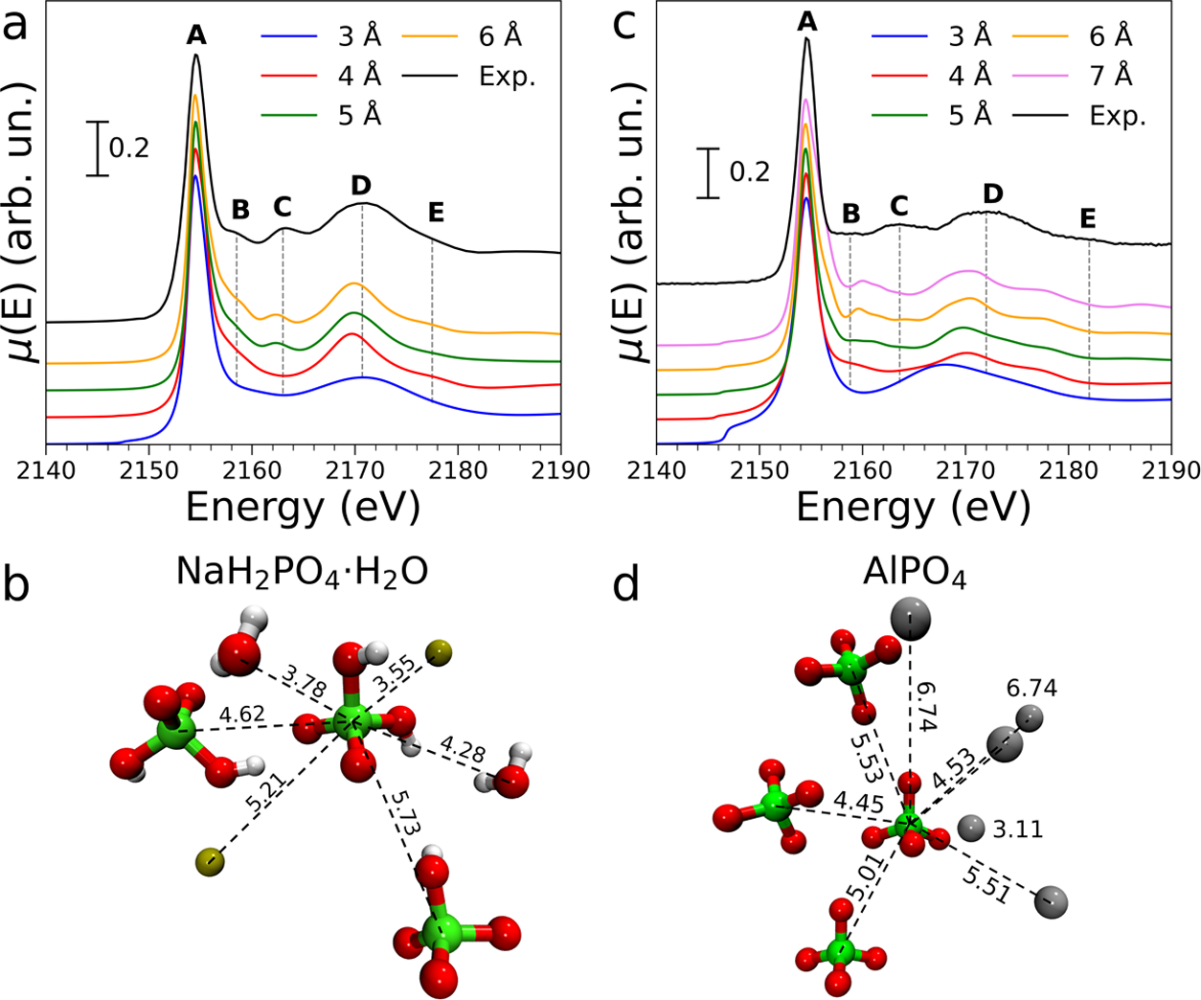
(a) Experimental P K-edge spectrum of NaH_2_PO_4_·H_2_O (black solid line) compared to the theoretical spectra calculated using an increasing cutoff radius. Gray dashed lines highlight the energy position of the observed features. (b) Depiction of the structure employed in the calculation of the NaH_2_PO_4_·H_2_O spectrum where distance-equivalent ions and water molecules have been omitted for clarity. Distances from the photoabsorber to the central atom of each ion or molecule (Å) are reported above the dashed lines. Color code: phosphorus, green; oxygen, red; sodium, olive; and hydrogen, white. (c) Experimental P K-edge spectrum of AlPO_4_ (black solid line) compared to the theoretical spectra calculated using an increasing cutoff radius. Gray dashed lines highlight the energy position of the observed features. (d) Depiction of the structure employed in the calculation of the AlPO_4_ spectrum where most distance-equivalent ions have been omitted for clarity. Distances from the photoabsorber to the central atom of each ion or molecule (Å) are reported above the dashed lines. Color code: phosphorus, green; oxygen, red; and aluminum, gray.

The experimental P K-edge XANES spectrum of NaH_2_PO_4_·H_2_O is shown in Figure 1a. This spectrum displays a sharp white line transition at 2154.6 eV (feature **A**), followed by two low-intensity peaks at 2158.5 (**B**) and 2162.6 eV (**C**), respectively, and a broad transition at 2170.6 eV (**D**) with a shoulder at 2177.6 eV (**E**). As the pre-edge region of the spectrum is featureless, no transitions to bound states are observed in this system. The crystallographic structure of NaH_2_PO_4_·H_2_O (space group P2_1_2_1_2_1_ (no. 19), *a* = 7.28 Å, *b* = 11.38 Å, and *c* = 6.61 Å) presents dihydrogen phosphate ions binding Na^+^ cations in a slightly distorted octahedral geometry.^38^ The dihydrogen phosphate ions are arranged in a tetrahedral geometry, with an average P–O bond distance of 1.51 Å, for the oxygen atoms possessing a partial negative charge, and on average equal to 1.58 Å for the oxygen atoms belonging to hydroxyl groups. The Na^+^ cations form face-sharing octahedra with the dihydrogen phosphate ions, where two oxygen atoms belonging to the hydroxyl groups are placed at an average 2.37 Å, distance from the Na^+^ cation, while three proton-free oxygen atoms of the H_2_PO_4_^–^ ion are found at an average bond distance of 2.49 Å. One water molecule completes each octahedron at a 2.47 Å, Na–O bond distance. All proton-free oxygen atoms and water molecules are shared between two neighboring sodium octahedra in an extended pattern.

In order to understand the structural contributions giving rise to the features observed in the experimental P K-edge XANES spectrum of NaH_2_PO_4_·H_2_O, as well as to estimate how far the structural sensitivity of P K-edge XANES extends, we calculated a series of theoretical spectra with the FDMNES program.^43^ The calculations have been performed including all atoms in the crystal structure of NaH_2_PO_4_·H_2_O within progressively increasing cutoff radii from the photoabsorber.^38^ The obtained theoretical spectra are shown in Figure 1a in comparison with the experimental data. When the cutoff radius is as low as 3 Å, the calculation is performed including only the first-shell oxygen atoms bound to the P photoabsorber that are arranged in a tetrahedral geometry, and two hydrogen atoms of the hydroxyl groups. One may note that, in the spectrum calculated using this cutoff radius, the white line shape matches that of the experimental spectrum and feature **D** appears, although the latter is slightly broader than in the experimental curve and shifted by about +1.0 eV with respect to its actual position. Upon increasing the radius to 4 Å, a shell of four Na^+^ ions at a distance of about 3.55 Å from the photoabsorber is included together with two water molecules at 3.78 Å, as shown in Figure 1b. The addition of these species in the calculation leads to a better agreement with the experimental data, as features **B** and **E** appear in the theoretical spectrum with a correct profile and the shape of feature **D** is significantly improved. The latter feature is also shifted to lower energy values (ca. −0.9 eV). Further increasing the cutoff radius to 5 Å including a farther shell of four dihydrogen phosphate ions at around 4.62 Å and three water molecules at about 4.28 Å results in feature **C** also being reproduced in the calculation at a correct intensity. The fact that species found at such distances have to be considered before feature **C** appears in the theoretical spectrum is not unexpected. It is in fact known that multiple scattering effects and second shell contributions can give rise to detectable features in the low-energy region of the XANES spectra after the main transition.^47^ As performing the calculation at a higher cutoff radius of 6 Å does not produce significant changes in the theoretical spectrum, it can be inferred that P K-edge XANES should be sensitive to the arrangement of atoms up to a distance of around 5 Å from the photoabsorber. This result will be further discussed and compared to calculations performed in different structures in the following sections.

In summary, both the relative intensities and the energy positions of all of the features found in the experimental spectrum of NaH_2_PO_4_·H_2_O are nicely reproduced employing a cutoff radius of 5 Å, proving the reliability of the theoretical framework used in the calculation. Only a slight energy shift of −0.6 eV for features **C** and ca. −0.9 eV for feature **D** is observed.

### AlPO_4_ System

The P K-edge XANES spectrum of AlPO_4_ is reported in Figure 1c: similarly to the XANES spectrum of NaH_2_PO_4_·H_2_O, the white line (feature **A**) is found at 2154.5 eV together with a low-intensity feature at 2158.8 eV (**B**), followed by two broader transitions centered at 2163.5 (**C**) and 2171.8 eV (**D**), respectively. The latter transition also presents a shoulder at 2181.7 eV (**E**).

It is important to note that the XANES spectra of NaH_2_PO_4_·H_2_O and AlPO_4_ present small but appreciable differences (see Figure S1), even though the local structure of phosphorus is very similar in the two systems. Phosphorus is in fact tetrahedrally coordinated with oxygen atoms in both the phosphate and dihydrogen phosphate ions. However, the crystal structures of these two compounds strongly differ in both counterion coordination and crystal system (octahedral and orthorhombic in NaH_2_PO_4_·H_2_O, tetrahedral and trigonal in AlPO_4_, respectively). AlPO_4_ crystallizes in the trigonal space group P3_2_21 (no. 154), with *a* = *b* = 5.00 Å, and *c* = 11.02 Å.^40^ Both phosphorus atoms and aluminum ions form regular tetrahedra, with average P–O and Al–O bond distances of 1.51 and 1.72 Å, respectively. Each phosphate anion is linked to four different Al^3+^ cations in an extended network.

The theoretical XANES spectrum of AlPO_4_ calculated including the phosphate ion (Figure 1c), specifically using a cutoff radius of 3 Å, only reproduces the white line shape (**A**) and feature **D**, the latter showing a high energy shift of −4.8 eV. A small feature in the absorption spectrum is also observed at about 2147.3 eV, likely due to a too large density of empty states computed by FDMNES in the pre-edge region. This is absent in the spectrum calculated with a 4 Å, cutoff, which includes a shell of four Al^3+^ ions directly bound to the central phosphate at a P–Al distance of 3.11 Å, (see Figure 1d), and the shift of feature **D** is lower (about −2.5 eV). Features **B** and **E** also arise in the theoretical curve at this cutoff value, although they are shifted by ca. −3.7 and ca. −4.2 eV, respectively. These two features can thus be ascribed to a contribution of the second coordination shell of phosphorus in the case of AlPO_4_ as well. If the cutoff radius is further increased to 5 Å, four phosphate ions at a distance of 4.45 Å and four Al^+3^ ions at 4.53 Å are included in the cluster. In this way, a bump appears in the energy region where feature **C** is found in the experimental spectrum. At a cutoff radius of 6 Å, a shell of six phosphate ions at a distance of 5.01 Å, together with a shell of four more phosphate ions at around 5.53 Å, and two Al^3+^ ions at 5.51 Å are included in the calculation. The intensity of feature **C** consequently increases, improving the agreement with the experimental data, and the shift of feature **D** is slightly lowered. However, features **B**, **C**, and **E** are shifted by about −1.6, −3.2, and −3.6 eV, respectively. The behavior of feature **C**, which was also observed to appear only in the spectra of clusters obtained with a cutoff radius above 5 Å, in NaH_2_PO_4_·H_2_O, suggests that it is associated with the single and multiple scattering paths extending to the outer shells of the photoabsorber. Increasing the cutoff to 7 Å does not produce a significant improvement in the agreement with the experimental curve.

Overall, the agreement between the experimental and theoretical spectra of AlPO_4_ is good, although a systematic shift in the energy position of the structural feature is observed. Both the shapes and relative intensities of the features match those found in the experimental data.

### α-Ti(HPO_4_)_2_·H_2_O System

**Figure 2:**
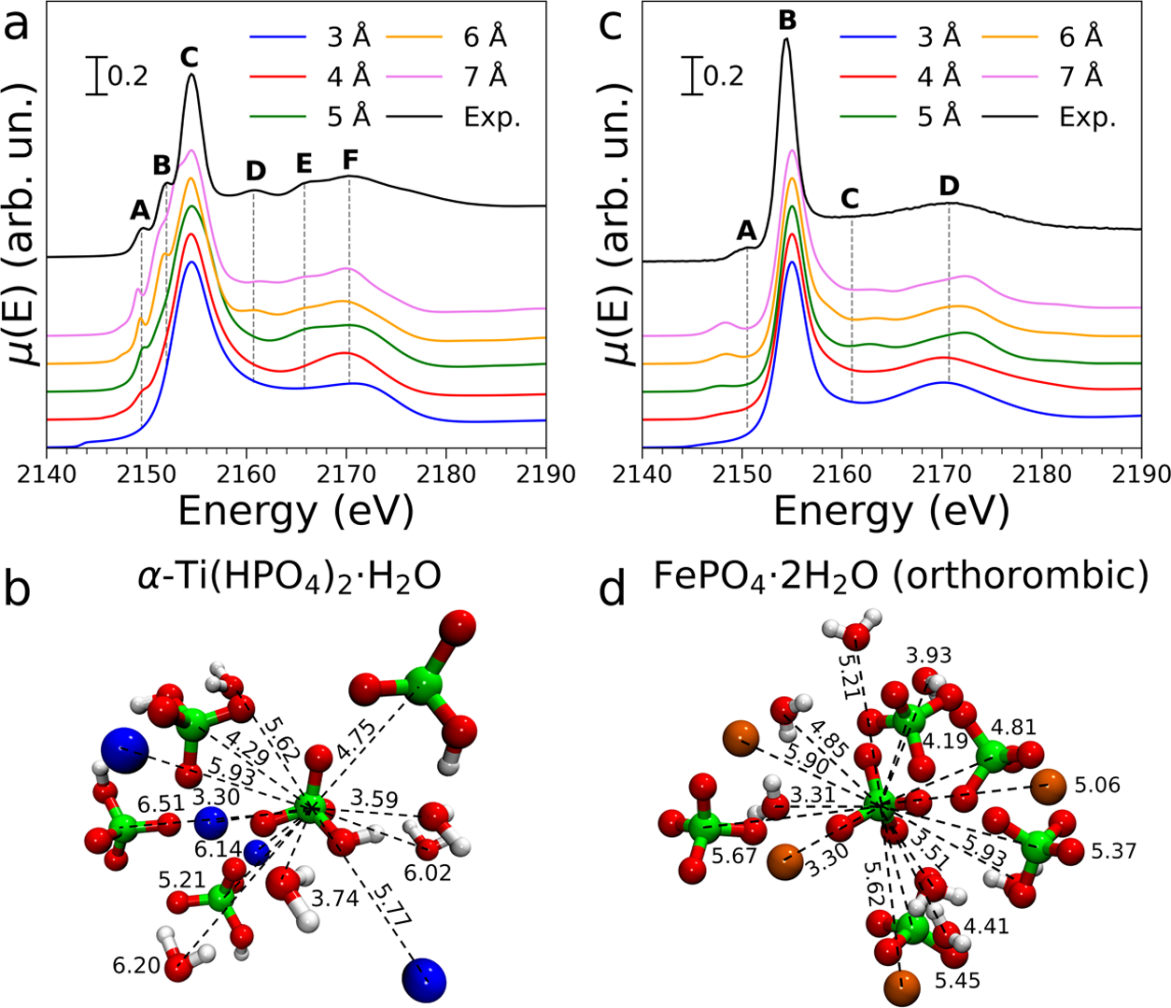
(a) Experimental P K-edge spectrum of α-Ti(HPO_4_)_2_·H_2_O (black solid line) compared to the theoretical spectra calculated using an increasing cutoff radius. Gray dashed lines highlight the energy position of the observed features. (b) Depiction of the structure employed in the calculation of the α-Ti(HPO_4_)_2_·H_2_O spectrum where distance-equivalent ions and water molecules have been omitted for clarity. Distances from the photoabsorber to the central atom of each ion or molecule (Å) are reported above the dashed lines. Color code: phosphorus, green; oxygen, red; titanium, blue; and hydrogen, white. (c) Experimental P K-edge spectrum of FePO_4_ (black solid line) compared to the theoretical spectra calculated using an increasing cutoff radius. Gray dashed lines highlight the energy position of the observed features. (d) Depiction of the structure employed in the calculation of the FePO_4_ spectrum where distance-equivalent ions have been omitted for clarity. Distances from the photoabsorber to the central atom of each ion or molecule (Å) are reported above the dashed lines. Color code: phosphorus, green; oxygen, red; iron, brown; and hydrogen, white.

In the P K-edge XANES spectrum of α-Ti(HPO_4_)_2_·H_2_O, shown in Figure 2a, two intense transitions to bound states are present in the pre-edge region. These fall at 2149.5 (feature **A**) and 2152.1 eV (**B**), respectively. The white line is located at 2154.5 eV (**C**), followed by a peak at 2160.8 eV (**D**) and a broad transition centered at 2170.3 eV (**F**) with a shoulder at 2165.8 eV (**E**). Pre-edge features such as those found in the XANES spectrum of this system are usually associated with transitions between core and valence orbitals localized on the photoabsorber, e.g., 1s → 3d transitions for 3d metals,^48^ but the complete absence of such features in the spectra of NaH_2_PO_4_·H_2_O and AlPO_4_, where phosphorus presents the same +5 oxidation state as α-Ti(HPO_4_)_2_·H_2_O, suggests a different scenario. The actual nature of these transitions is not trivial to understand and will therefore be further discussed in the following sections.

The crystal structure of α-Ti(HPO_4_)_2_·H_2_O (space group P2_1_/c (no. 14), *a* = 8.61 Å, *b* = 4.99 Å, *c* = 16.15 Å, α = γ = 90°, β = 110.2°) resembles the structure of NaH_2_PO_4_·H_2_O.^39^ The average P–O bond distance in the tetrahedral monohydrogen phosphate anions is 1.55 Å, for proton-free oxygen atoms and 1.56 Å, for hydroxyl groups, while the Ti–O bond distance is 1.92 Å. Monohydrogen phosphate binds Ti^4+^ cations in an octahedral geometry with all oxygen while leaving the hydroxyl groups free to form hydrogen bond interactions with water molecules, which are intercalated between titanium phosphate layers in a typical clay-like structure.

When the theoretical spectrum of α-Ti(HPO_4_)_2_·H_2_O is calculated using only the monohydrogen phosphate structure (cutoff radius 3 Å, Figure 2a), only the white line shape and the broad feature **F**, which is again confirmed to be associated with the first coordination shell of phosphorus, are reproduced. The latter feature is however shifted by about ∼+2.0 eV with respect to the energy position found in the experimental curve. A small bump is observed around 2143.5 eV in the theoretical spectrum as in the case of AlPO_4_. Upon increasing the cutoff radius to 4 Å, three Ti^4+^ ions bound to the central monohydrogen phosphate ion are included in the calculation, as well as three water molecules at a distance of around 3.74 Å, (see Figure 2b). It can be noted that this determines an improvement in the agreement of feature **F** with the experimental data as it is only shifted by ca. −0.4 eV if compared to the experimental curve. Additionally, at this cutoff radius feature **A**, which is a pre-edge peak and corresponds to a transition to bound states, appears in the experimental spectrum at the correct energy position. Calculating the theoretical spectrum of α-Ti(HPO_4_)_2_·H_2_O with a cutoff radius of 5 Å, namely including a shell of seven monohydrogen phosphate ions at distances ranging from 4.29 to 4.75 Å, results in the appearance of feature **E** at the correct energy position. In a similar manner, at a cutoff radius of 6 Å, features **B** and **D** appear in the theoretical curve and they are aligned with the features observed in the experimental data. This value of the cutoff radius implies that a shell of five more monohydrogen phosphate ligands at a distance of around 5.21 Å, a water molecule at 5.62 Å, and two Ti ^4+^ ions at 5.77 and 5.93 Å, respectively, are included in the cluster. Altogether, these findings suggest that features **A** and **B** are due to Ti ^4+^ cations belonging to different coordination shells, the former being related to ions more tightly bound to monohydrogen phosphate. The theoretical spectrum does not benefit from the increase of the cutoff radius up to 7 Å, as only small differences between this spectrum and the one calculated with a cutoff radius of 6 Å, are observed. The obtained agreement between the experimental and theoretical curves is very good, as all features present in the experimental curve are reproduced at accurate relative intensities and energy positions. Since convergence of the theoretical spectra is achieved at a cutoff radius of 6 Å, in agreement with the result obtained for NaH_2_PO_4_·H_2_O and AlPO_4_, it is confirmed again that P K-edge XANES is sensitive to the molecular structure of phosphorus compounds up to 6 Å, from the photoabsorber. Further, additional proof of the high structural sensitivity provided by P K-edge XANES can be evinced by looking at the spectra of NaH_2_PO_4_·H_2_O and α-Ti(HPO_4_)_2_·H_2_O (Figure S1). These compounds contain similar phosphorus species, but their XANES spectra present marked differences besides the pre-edge transitions observed for α-Ti(HPO_4_)_2_·H_2_O.

We have to remark that, unlike the calculations performed for NaH_2_PO_4_·H_2_O, AlPO_4_, and FePO_4_·2H_2_O, the theoretical spectrum of α-Ti(HPO_4_)_2_·H_2_O had to be calculated at the MS level to obtain a better agreement with the experimental data. In fact, in the spectrum of the latter structure calculated at the FDM level (see Figure S2), feature **A** is shifted by ∼+ 1.7 eV if compared to the calculation performed at the MS level, and feature **B** is not reproduced. Further, the white line shape is split into two components, and feature **D** is shifted toward lower energies by ∼1.5 eV.

### FePO_4_·2H_2_O System

The P K-edge XANES spectrum of FePO_4_·2H_2_O (Figure 2c) also shows a pre-edge transition at 2150.1 eV (feature **A**), while the white line falls at 2154.2 eV (**B**). At higher energies, a main broad peak centered at 2171.0 eV is observed (**D**) with a shoulder at 2162.0 eV (**C**). The fact that this spectrum is vastly different from that of AlPO_4_, which also presents phosphate as a counterion, is yet another proof of the high structural sensitivity of P K-edge XANES.

It has to be remarked, however, that FePO_4_·2H_2_O is a polymorphic mineral that exists in two nearly isostructural, but not isomorphous, crystal phases. These show identical connectivity but different spatial arrangement of the phosphate and iron ions.^41^ The crystal structure of orthorhombic FePO_4_·2H_2_O (space group Pbca (no. 61), *a* = 9.87 Å, *b* = 10.10 Å, *c* = 8.70 Å) presents tetrahedral phosphate ions bound to octahedral Fe^3+^ ions in a corner sharing pattern.^41^ Each Fe^3+^ octahedron also contains two water molecules that interact with the phosphate atoms through hydrogen bonds. The average P–O bond distance is 1.53 Å, while the Fe–O bond distance is 1.97 Å on average for the phosphate ions and 2.07 Å, for water.

**Figure 3:**
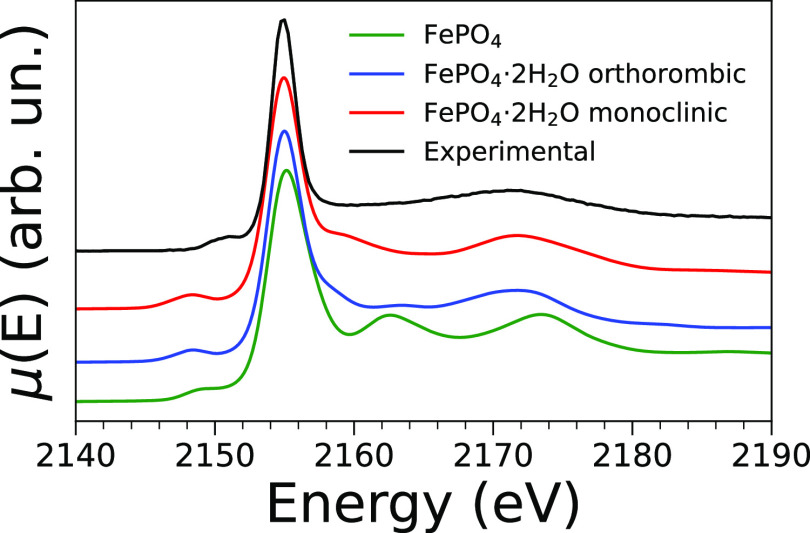
Comparison between the experimental P K-edge XANES spectrum of FePO_4_·2H_2_O with the theoretical spectra of FePO_4_·2H_2_O (6 Å, cutoff, orthorhombic phase), FePO_4_·2H_2_O (6 Å, cutoff, monoclinic phase), and FePO_4_ (5 Å, cutoff, anhydrous).

The theoretical P K-edge spectrum of a phosphate ion in orthorhombic FePO_4_·2H_2_O (Figure 2c, 3 Å, cutoff radius) only reproduces the white line shape of the experimental curve and feature **D**, which is shifted to lower energies by about 1.1 eV with respect to the experimental data. Figure 2d shows a depiction of the cluster employed in the calculations. At a cutoff radius of 4 Å, the cluster includes four additional Fe^3+^ ions directly bound to the phosphate ion, placed at a distance of 3.30 Å from the central phosphorus atom. Nine water molecules found at distances between 3.31 and 3.93 Å are also included in the cluster. Surprisingly, this modification of the cluster has little effect on the theoretical curve, where feature **D** is shifted to ca. −1.5 eV and no new features are observed besides a bump appearing at about 2148.3 eV in the pre-edge region. The latter can be attributed once more to a forbidden transition to bound states possessing d character, arising from orbital mixing between phosphorus and iron (vide infra) since it only appears when Fe^3+^ ions are considered in the calculation. Increasing the cutoff radius to 5 Å, adds one phosphate ion at 4.19 Å, and two more at 4.81 Å, to the cluster, as well as two water molecules placed at 4.41 and 4.85 Å, respectively. In parallel to what has been presented in the previous sections, feature **C** appears in the spectrum calculated using the cluster mentioned above (∼+1.4 eV from the expected energy position); therefore, it can be linked to the contribution of molecules placed in farther coordination shells of phosphorus. In an attempt to further separate the contribution of the long-distance water and phosphate ions into shaping feature **C**, we calculated a series of spectra using different clusters obtained by removing these species one at a time from the cluster cut at 5 Å, (see Figure S3). Remarkably, feature **C** is present in each of the calculated spectra, showing that the contribution of water and phosphate ions to the multiple scattering processes is somewhat similar and cannot be easily singled out. The intensity of feature **A** is also slightly increased and feature **D** shifts to ∼+1.1 eV. When the cutoff radius is brought up to 6 Å, three Fe^3+^ ions (placed at 5.06, 5.45, and 5.90 Å, respectively), five phosphate ions (placed between 5.37 and 5.67 Å), and seven water molecules (5.21–5.93) are included in the cluster. This further improves the agreement between the experimental and theoretical curves, as feature **A** is more well defined and its position is shifted to ca. −2.7 eV. It can be noted that convergence is achieved at 6 Å, in this compound as well, since increasing the cutoff radius to 7 Å, does not have an appreciable impact on the theoretical curve. Overall, the theoretical spectrum of orthorhombic FePO_4_·2H_2_O presents nice agreement with the experimental curve, as the profile, energy position, and relative intensity of each spectral feature match well. However, the polymorphic nature of FePO_4_·2H_2_O imposes a further step in the theoretical analysis, which has to be performed on the monoclinic phase as well. We also have to note that no information on the phase composition of commercial FePO_4_·2H_2_O is available, making a comparison between the two possible phases mandatory. See Section 1.1 in the Supporting Information for a detailed comment on the crystal structure and theoretical spectra of monoclinic FePO_4_·2H_2_O, and Figure S4a,b for a comparison of the theoretical spectra with the experimental one as well as a depiction of the cluster used in the calculations, respectively. Briefly, the theoretical spectrum of monoclinic FePO_4_·2H_2_O presents slightly less good agreement with the experimental data if compared to that of the orthorhombic phase, as feature **C** is too intense and shifted close to the white line transition. Such difference must stem from the three-dimensional arrangement of the phosphate ions in monoclinic FePO_4_·2H_2_O, which is more symmetrical. The probed cluster of orthorhombic FePO_4_·2H_2_O instead presents a higher degree of disorder, yielding less intense spectral features. A comparison between the theoretical spectra of each phase and the experimental one is shown in Figure 3. Although the presence of the monoclinic phase cannot be completely ruled out based on the observed spectral variations, it is reasonable to assume that orthorhombic FePO_4_·2H_2_O is the main component of the analyzed sample. We therefore deduce that the P K-edge XANES technique is even sensitive to structural differences between crystal phases of the same chemical species that are identical in chemical connectivity, but not in crystal structure.

To further explore the sensitivity of the technique toward slightly different forms of the same compound, the theoretical spectra of anhydrous FePO_4_ (orthorhombic) have also been computed. A detailed comment on these calculations is provided in Section 1.2 in the Supporting Information, while the spectra are compared to the experimental spectrum of FePO_4_·2H_2_O in Figure S5a, and the cluster employed in the calculations is shown in Figure S5b. Figure 3 also shows the comparison between the theoretical spectrum of FePO_4_ and the experimental spectrum of FePO_4_·2H_2_O. Although the two are not drastically different, it is evident that the intensity of feature **C** in the former is too intense. This finding shows that P K-edge XANES can also distinguish between hydrated and dehydrated forms of the same material that present similar crystal structures.

### Contribution of Hydrogen Atoms in the XANES Calculations

The contribution of hydrogen atoms to P K-edge XANES calculations was also investigated. Although this element is often neglected in XANES calculations because of its low expected contribution to the overall potential, clearly detectable hydrogen contributions have been observed in the K-edge spectra of TM aqua ions, where the EXAFS signal could only be properly reproduced if the first-shell H_2_O protons were considered.^49^

Monohydrogen and dihydrogen phosphate ions were cut from the crystal structures of NaH_2_PO_4_·H_2_O and α-Ti(HPO_4_)_2_·H_2_O and theoretical P K-edge XANES spectra were then calculated from these structures after removing all hydrogen atoms (which is equivalent to converting each structure into a phosphate ion). These spectra have been compared to the theoretical spectra of their protonated counterparts (Figure S6a,b, respectively), revealing noticeable contributions of the protons. In the theoretical spectrum of the phosphate ion obtained from NaH_2_PO_4_·H_2_O (Figure S6a), the first post-edge feature is split into two peaks, at variance with what is observed in the theoretical spectrum calculated from the real structure. Additional features in the absorption coefficient are also observed in the pre-edge region for both systems and they can be ascribed to a charge density imbalance on the oxygen atoms bound to the central phosphorus that may arise if hydrogen atoms are not considered in the calculation.

Conversely, the theoretical XANES spectrum of the proton-free phosphate obtained from α-Ti(HPO_4_)_2_·H_2_O (Figure S6b) shows modest, but still noticeable, differences from that of the respective monohydrogen phosphate structure. In the former, the white line transition is unexpectedly broad, and the post-edge feature is shifted to lower energies by ∼1.0 eV. Both effects can be ascribed to the previously discussed charge imbalance of the cluster, with the deprotonated phosphate extracted from α-Ti(HPO_4_)_2_·H_2_O exhibiting a potential closer to that observed in the experimental data due to the removal of only one hydrogen atom, whereas the removal of two hydrogen atoms in the phosphate extracted from NaH_2_PO_4_·H_2_O resulted in a potential that deviates to a higher extent from the experimental spectrum.

Collectively, these results highlight how hydrogen atoms placed at close distances from the photoabsorber provide a detectable impact on P K-edge XANES calculations despite their expected contribution to the overall potential being limited.

### Nature of the Pre-Edge Transitions

As mentioned above, pre-edge features in K-edge X-ray absorption spectra are commonly assigned to transitions that promote 1s electrons to empty valence states localized on the absorber atom. In the case of third-row element X-ray spectra, and in particular for chlorine and sulfur XAS, these states have also been shown to arise because of covalent mixing between metal and ligand orbitals.^45,46,50−53^ Pre-edge peaks stemming from this covalent mixing can therefore be observed if the empty (f or d) metal orbitals exhibit marked ligand p character.^45,50^ Similarly, pre-edge transitions have been diffusely observed in a number of P K-edge spectra of TM phosphates,^2,8,22,36,54,55^ with the exception of Zn phosphates.^55^ Useful qualitative and quantitative information can be obtained by careful analysis of these pre-edge features, as they seem to vary between the spectra of phosphorus compounds containing different TMs. Suitable fingerprints for the identification of each metal are therefore provided by these transitions. In the case of iron phosphates, pre-edge transitions have even been found to correlate with the iron oxidation state.^6^ However, according to classical ligand-metal field theory, all valence electrons are employed in chemical bonds in phosphorus (V) compounds (those considered in this work) and none of such empty states are available. The XANES spectra of NaH_2_PO_4_·H_2_O and AlPO_4_, in addition to that of LaPO_4_ (see Figure S1), show in fact no pre-edge transitions at all. This preliminary finding supports the hypothesis that the pre-edge features observed in the XANES spectra of α-Ti(HPO_4_)_2_·H_2_O and FePO_4_·2H_2_O arise from a contribution of the Ti^4+^ and Fe^3+^ ions. Although these are not directly bound to phosphorus in the investigated systems, the experimental evidence still suggests that a certain degree of covalency is present, leading to the frontier orbitals involved in the pre-edge transitions. We can thus infer that such covalent bonding is not observed in the sodium, aluminum, and lanthanum compounds. To further investigate this matter, we calculated both the total and angular momentum resolved DOS for all atoms in α-Ti(HPO_4_)_2_·H_2_O and FePO_4_·2H_2_O, aiming to single out the contribution of metal species in the XANES spectra.

**Figure 4:**
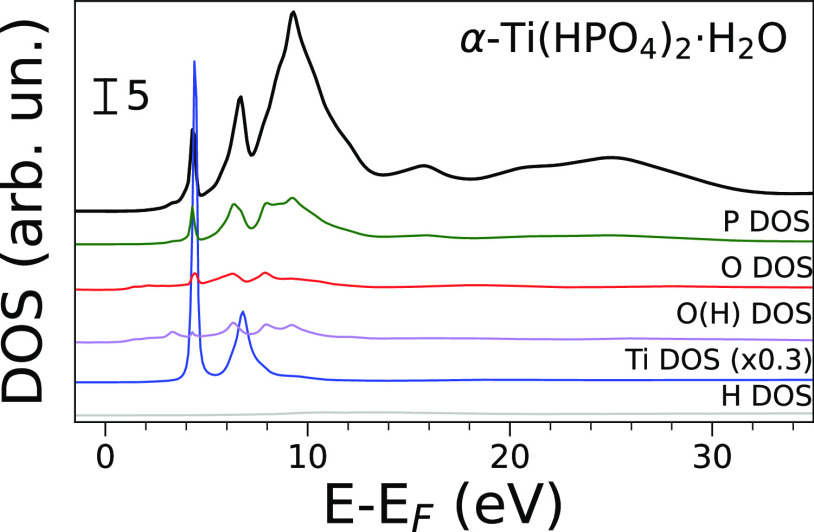
Theoretical spectrum of α-Ti(HPO_4_)_2_·H_2_O (black solid line, no convolution) and density of states (DOS) of P (green), O (red for proton-free O, purple for OH), Ti (blue), and H (gray) calculated using a cutoff radius of 6 Å. The theoretical spectrum and Ti DOS have been scaled for better comparison.

The calculated atom-projected DOS of α-Ti(HPO_4_)_2_·H_2_O, compared to the theoretical XANES spectrum of this compound, is shown in Figure 4. Remarkably, the titanium DOS of this system is featureless in the XANES region except for two sharp and intense peaks at ∼5 and ∼7 eV above the Fermi level. These are precisely aligned with the pre-edge features in the theoretical spectrum of this compound (Figure 2c, features **A** and **B**). Such a result unambiguously indicates that the presence of titanium gives rise to molecular orbitals presenting a high degree of mixing between phosphorus and titanium orbitals. The Ti^4+^ ion possesses in fact both a high positive electric charge and a formal 3d^0^ electronic configuration, making it extremely prone to interact with oxygen donor species (it has high Lewis acidity) and therefore to orbital mixing. Additionally, the phosphorus DOS presents intense peaks aligned with those found in the titanium DOS, confirming that these states are localized on both atoms. This result is quite interesting, since as previously stated titanium and phosphorus atoms are not directly bound in the α-Ti(HPO_4_)_2_·H_2_O structure but are instead bridged by oxygen. Such a high degree of covalency is consequently not straightforward, but a similar phenomenon has been observed in the FeTiO_3_ system.^56^ Low-intensity peaks in the oxygen DOS aligned with those found in the titanium DOS confirm its role in mediating the mixing process of titanium and phosphorus states, while the hydrogen DOS shows an almost flat profile. Additionally, we calculated the projected DOS phosphorus and titanium in clusters obtained from the α-Ti(HPO_4_)_2_·H_2_O crystal structure using increasing cutoff radii. This DOS analysis, shown in Figure S7a,b, confirms the previous hypothesis that feature **B** in the XANES spectrum of α-Ti(HPO_4_)_2_·H_2_O (Figure 2a) is due to the shell of Ti^4+^ ions placed at 5.77 Å from the photoabsorber. In fact, both the phosphorus and titanium DOS only show the intense peaks corresponding to feature **B** when the cluster radius is increased to 6 Å, that is when the aforementioned long-distance Ti^4+^ ions are included in the cluster. We can therefore attribute this transition to a multiple scattering resonance involving a delocalized many-body final state, where the photoelectron reaches the outer shell of Ti^4+^ ions. A similar phenomenon has been observed by Soldatov et al. in the XANES spectrum of CeO_2_.^57^ In order to single out the role of s, p, and d orbitals in the molecular orbitals, we also calculated the angular momentum resolved projected DOS for phosphorus and titanium in α-Ti(HPO_4_)_2_·H_2_O. The resulting DOS calculation, which is reported in Figure S8, clearly shows that only titanium d orbitals mix with the phosphorus orbitals. In fact, the titanium s- and p-DOS are completely flat while its d-DOS presents the two peaks also observed in the total DOS shown in Figure 4. From the point of view of ligand-metal field theory, the two peaks can be assigned to the well-known t_2__g_ and e_g_ sets of molecular orbitals. Conversely, both the phosphorus s- and p-DOS overlap with the titanium d-DOS. The observed pre-edge peaks can thus be assigned to a transition between the phosphorus 1s orbital and valence (s and p) bound states, where the latter possess a high degree of d character provided by the contribution of titanium. Finally, the results presented in Figure S8 also allow us to assign the white line feature in the P K-edge spectrum of α-Ti(HPO_4_)_2_·H_2_O (Figure 2c, feature **C**). Since the phosphorus s DOS is negligible at the white line energy while the p-DOS shows instead a well-defined peak, we assign the white line to a transition from the phosphorus 1s orbital to empty p orbitals beyond the Fermi level. This result is consistent with previous investigations based on molecular orbital calculations.^30^ To further corroborate our hypotheses regarding the Ti contribution, we performed additional P K-edge XANES and DOS calculations replacing Ti^4+^ ions with Na^+^ ions in the crystal structure of α-Ti(HPO_4_)_2_·H_2_O, obtaining a purely hypothetical anionic structure that we name α-Na(HPO_4_)_2_·H_2_O. Figure S9a shows the comparison between the theoretical XANES spectra of α-Ti(HPO_4_)_2_·H_2_O and α-Na(HPO_4_)_2_·H_2_O. Strikingly, one may observe that the theoretical spectra of the two structures are almost identical, especially as far as the white line shape and post-edge features are concerned, but the spectrum of α-Na(HPO_4_)_2_·H_2_O presents no features in the pre-edge region at all. This can be regarded as further proof that the transitions observed in the XANES spectrum of α-Ti(HPO_4_)_2_·H_2_O are due to covalent mixing between titanium d and phosphorus s/p states, which cannot happen in α-Na(HPO_4_)_2_·H_2_O due to the absence of accessible d states in sodium ions. In fact, the atom-projected DOS of α-Na(HPO_4_)_2_·H_2_O (Figure S9b) is completely flat for sodium in the observed region, while for all other atoms, it closely resembles that of α-Ti(HPO_4_)_2_·H_2_O except the pre-edge transitions.

**Figure 5:**
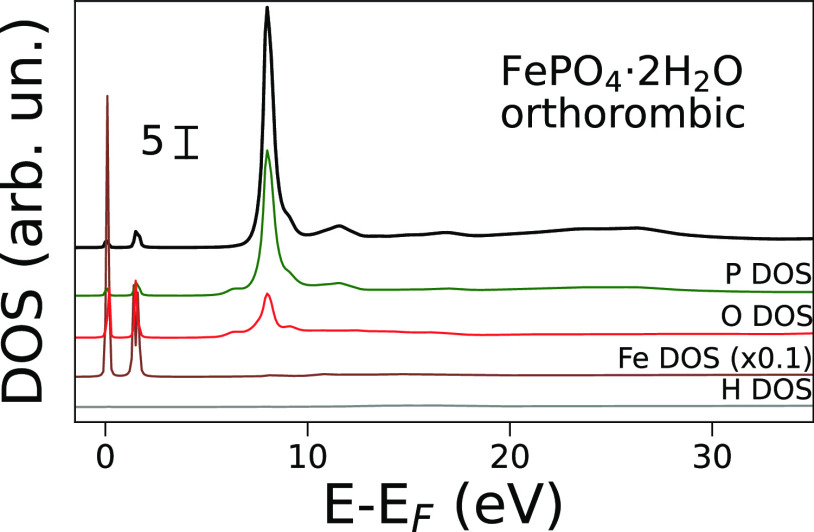
Theoretical P K-edge XANES spectrum of orthorhombic FePO_4_·2H_2_O (black solid line, no convolution) and density of states (DOS) of P (green), O (red), and Fe (brown) calculated using a cutoff radius of 6 Å. The theoretical spectrum has been scaled for better comparison.

A projected DOS analysis of orthorhombic FePO_4_·2H_2_O (Figure 5) led to similar conclusions as those discussed for α-Ti(HPO_4_)_2_·H_2_O. In this case, the Fe DOS presents two sharp and intense sets of peaks placed right at and above the Fermi level, a result consistent with the electron conductivity properties shown by this compound.^58^ These peaks can also be attributed to t_2g_ and e_g_ molecular orbitals and they align well with those observed in the phosphorus and oxygen DOS, indicating that also in FePO_4_·2H_2_O phosphorus and iron states mix to some degree in a process mediated by oxygen. For this system, the projected phosphorus and iron DOS calculated at increasing cutoff radii (see Figure S10) show that the bump observed in the theoretical spectrum calculated at a 4 Å, cutoff (Figure 2, feature **A**) is indeed already due to the mixing of iron states with phosphorus ones. Higher shell Fe^3+^ ions also contribute to this feature as the Fe DOS intensity rises when expanding the cluster. A subsequent angular momentum resolved DOS analysis (see Figure S11) again showed that only iron d orbitals participate in the covalent interaction with phosphorus s and p orbitals, giving rise to mixed states with d character that are involved in said pre-edge transition. Consequently, the pre-edge transition can again be assigned to the promotion of 1s electrons to valence-bound states of metal character in the case of orthorhombic FePO_4_·2H_2_O. In addition, the analysis shown in Figure S11 reveals that the final states involved in the white line transition in the XANES spectrum of this mineral are of p character as well. For the sake of comparison, the same analyses have been performed for monoclinic FePO_4_·2H_2_O and anhydrous FePO_4_ (Figures S12 and S13, respectively), showing practically identical results as those discussed for orthorhombic FePO_4_·2H_2_O.

Finally, the projected DOS of NaH_2_PO_4_·H_2_O and AlPO_4_ are shown in Figures S14 and S15, respectively, for completeness. As expected, the NaH_2_PO_4_·H_2_O DOS does not show any structure in the pre-edge region and only presents broad peaks above the edge. The same holds for AlPO_4_, where the DOS of aluminum has an even lower intensity. For each structure, the phosphorus, oxygen, and hydrogen DOS show a similar behavior as that observed for α-Ti(HPO_4_)_2_·H_2_O and FePO_4_·2H_2_O. In both cases, the final states involved in the white line transition possess a strong p character, in line with the results discussed for α-Ti(HPO_4_)_2_·H_2_O and FePO_4_, although they also present a modest s character in AlPO_4_.

## Conclusions

Theoretical P K-edge XANES spectra of NaH_2_PO_4_·H_2_O, AlPO_4_, α-Ti(HPO_4_)_2_·H_2_O, and FePO_4_·2H_2_O, displaying excellent agreement with the experimental data, have been presented. It has been shown how different coordination spheres yield separate features in the XANES signal, and it was found that the technique is highly sensitive to the structural arrangement of chemical species found at a distance of up to 6 Å, from the photoabsorber. The XANES spectra of the investigated compounds present in fact marked differences even though the first-shell coordination of phosphorus is nearly the same in all structures. Our results also proved that the XANES technique possesses impressive structural sensitivity to slightly different forms of the same compound, allowing us to assign the orthorhombic phase of FePO_4_·2H_2_O as the main component of the investigated sample. Additionally, it has been shown how DOS calculations can provide a rationalization of pre-edge features observed in the spectra of TM phosphates. We determined that these transitions arise because of covalent mixing between phosphorus s and p and TM d states in α-Ti(HPO_4_)_2_·H_2_O and FePO_4_·2H_2_O.

We think that our findings can pave the way for a more widespread use of quantitative theoretical calculations in the analysis of phosphorus XANES, acting as a solid background for future theoretical investigations. The present analysis may lead to the development of advanced phosphorus speciation analysis protocols supported by theoretical simulations, with the significant advantage of not having to rely on an existing database of standards.

## Supporting Information Available

Additional comments on the theoretical P K-edge spectra and supplementary figures and tables (PDF)
