# Supplementary material for: P K-Edge XANES Calculations of Mineral Standards: Exploring the Potential of Theoretical Methods in the Analysis of Phosphorus Speciation
Source: Inorg Chem. 2023 Jun 29;62(28):11188–98. doi: 10.1021/acs.inorgchem.3c01346 (PMC10354742; doi:10.1021/acs.inorgchem.3c01346)
Supplement: Supplementary file 1 — ic3c01346_si_001.pdf [file ic3c01346_si_001.pdf]

Supporting Information: P K-edge XANES Calculations of Mineral Standards: Exploring the Potential of Theoretical Methods in the Analysis of Phosphorus Speciation

Alessandro Tofoni,[†] Francesco Tavani,[†] Ingmar Persson,[‡] and Paola D'Angelo[*,†]

†*Department of Chemistry, Sapienza University of Rome, P.le A. Moro 5, 00185 Rome, Italy*

‡*Department of Molecular Sciences, Swedish University of Agricultural Sciences, P.O. Box 7015, SE-750 07 Uppsala, Sweden*

E-mail: p.dangelo@uniroma1.it

Contents

1 Supplementary Comments on the Theoretical XANES Spectra S-2

1.1 $FePO_4{\cdot}2H_2O$ (monoclinic) S-2

1.2 $FePO_4$ (anhydrous) S-3

2 Supplementary Figures (Figures S1 – S15) S-4

3 Supplementary Tables (Table S1) S-14

References S-15

1 Supplementary Comments on the Theoretical XANES Spectra

1.1 $FePO_4{\cdot}2H_2O$ (monoclinic)

The monoclinic phase of $FePO_4{\cdot}2H_2O$ (space group $P2_1/n$ (no. 14), a=5.31 Å, b=9.75 Å, c=8.68 Å, $\beta = 90.2°$) presents the same connectivity as the orthorombic one, with $Fe^{III}O_6$ octahedra and PO_4^{3-} tetrahedra forming a three-dimensional network.[S1] However, in this phase the relative orientations of the polyhedra differ from the orthorombic phase, as many layers of the three-dimensional network are aligned along the c axis. The average P-O bond distance is 1.53 Å in the phosphate ions, while the average Fe-O bond distance is 1.96 Å for the phosphate ions and 2.04 Å for water.

The theoretical spectrum of monoclinic $FePO_4{\cdot}2H_2O$ calculated using a cutoff radius of 3 Å (phosphate ion only) is shown in figure S4a. In this spectrum, only the whiteline shape and feature **D** are evident, and the latter is centered at a slightly lower energy (~-0.1 eV) with respect to the experimental data. Increasing the cutoff to 4 Å includes four additional Fe^{3+} ions (two at 3.37 Å and two at 3.27 Å) and eight water molecules at various distances between 3.19 and and 3.69 Å. The cluster employed in the calculation is shown in Figure S4b. At this value of the cutoff radius, features **A** and **C** appear in the theoretical spectrum, the former being shifted by ~-2.7 eV. The fact that feature **A** only appears when Fe^{3+} ions are included in the cluster suggests that it is due a transition towards Fe-localized bound states (see discussion in the main text). If the cutoff radius is further increased to 5 Å, only one additional phosphate ion placed at 4.42 Å and a water molecule at 4.97 Å are considered in the cluster. The resulting spectrum presents the same features as the one calculated using a cutoff radius of 4 Å. Nevertheless, the intensity of feature **A** is slightly increased and

feature **D** shifts. If the theoretical spectrum is calculated imposing a cutoff radius of 6 Å, the cluster includes two Fe^{3+} ions placed at 5.38 Å and 5.42 Å, as well as eight phosphate ions at distances between 5.38 and 5.42 Å and eight water molecules placed around 5.29 and 5.83 Å from the central phosphorus atom. In this way, the agreement between the theoretical curve and the experimental one is improved, as the intensity of feature **A** increases. An intensity decrease is also observed for feature **C**, which in this case is shifted by about 1.9 eV. The shift of feature **D** is also lowered to ∼-0.6 eV. Increasing the cutoff radius to 7 Å does not alter the theoretical curve in a significant manner.

1.2 $FePO_4$ (anhydrous)

In the crystallographic structure of anhydrous $FePO_4$ (space group Pnma (no. 62), a=9.81 Å, b=5.78 Å, c=4.78 Å, $\alpha=\beta=\gamma=90°$),[S2] the average P-O bond distance is 1.52 Å. Tetrahedral phosphate anions coordinate Fe^{3+} cations forming edge-sharing octahedra with a mean Fe-O bond distance of 2.05 Å, where two out of four phosphate oxygen atoms bridge two different iron atoms in parallel layered networks that expand along the a and c crystallographic axes. The remaining two oxygen atoms only bind one Fe^{3+} ion each, connecting the octahedra along the b crystallographic axis while acting as spacers between different iron layers.

Figure S5a shows the theoretical spectrum of anhydrous $FePO_4$ calculated by taking into account only the phosphate ligand (2.5 Å cutoff radius). The cluster employed in the calculation is shown in Figure S5b. Feature **D**, again proved to be associated with the first-shell oxygen atoms bound to the photoabsorber, is present in this spectrum and it is shifted by ∼-2.4 eV. Including the closest (2.79 Å) Fe^{3+} ion bound to the phosphate group, which is equivalent to increasing the cutoff radius to 3 Å, results in the appearance of feature **C** in the spectrum although it it shifted by about +3.0 eV. A calculation where the cutoff radius is increased to 4 Å includes a shell of four more Fe^{3+} ions at about 3.19 Å and two more phosphate ions at 3.56 Å. At this cutoff radius, the intensity and energy shift of features **C** and **D** are improved significantly. Increasing the radius again to 5 Å adds six phosphate

ions at a distance of about 4.78 Å and two Fe^{3+} ions at 4.92 Å to the cluster employed in the calculation. With this cluster, the intensity of feature **A** is more correctly reproduced, although its energy position does not change appreciably, and features **C** and **D** are further shifted to. Increasing the cutoff radius to 6 Å does not significantly alter the theoretical spectrum.

2 Supplementary Figures (Figures S1 – S15)

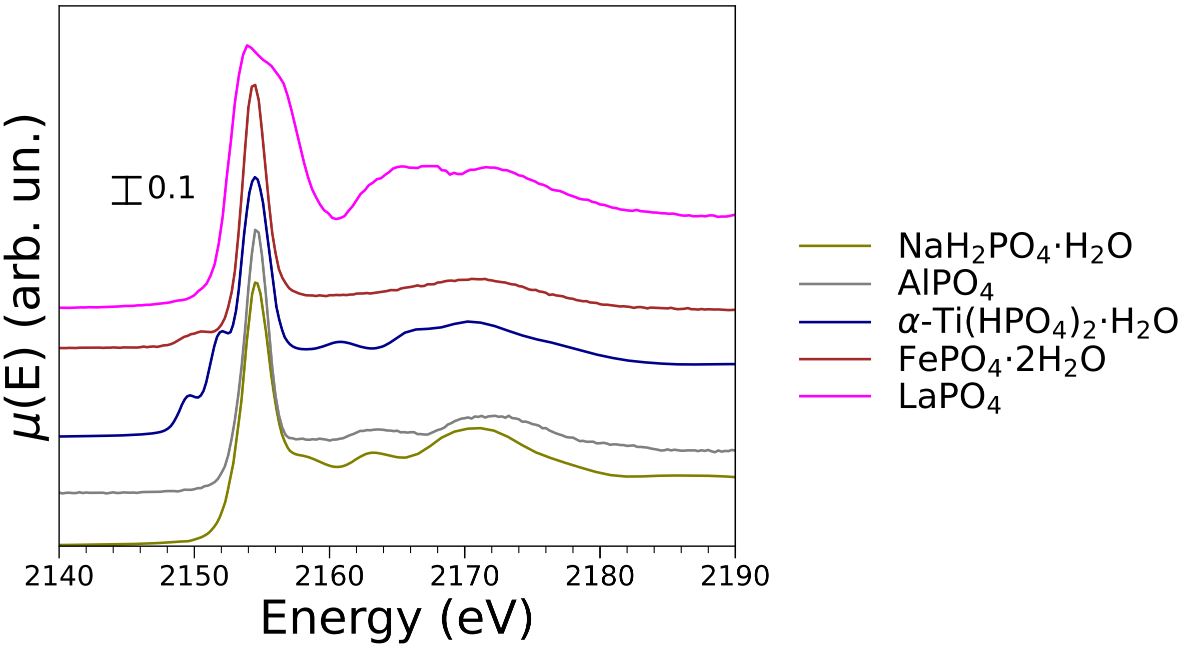

Figure S1: Experimental P K-edge XANES spectra of $LaPO_4$, $NaH_2PO_4 \cdot H_2O$, $AlPO_4$, α-$Ti(HPO_4)_2 \cdot H_2O$ and $FePO_4 \cdot 2H_2O$.

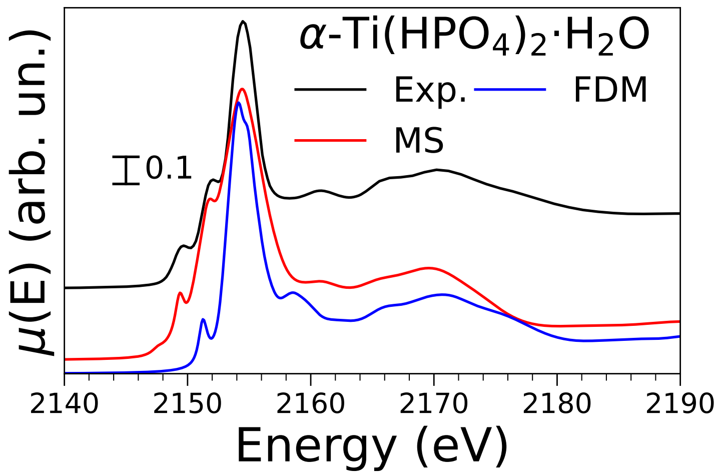

Figure S2: Theoretical P K-edge XANES of α-Ti$(HPO_4)_2 \cdot H_2O$ calculated using a cluster radius of 6 Å at the multiple scattering theory (MS, red curve) and finite differences method (FDM, blue curve) levels of theory compared to the experimental spectrum (black curve).

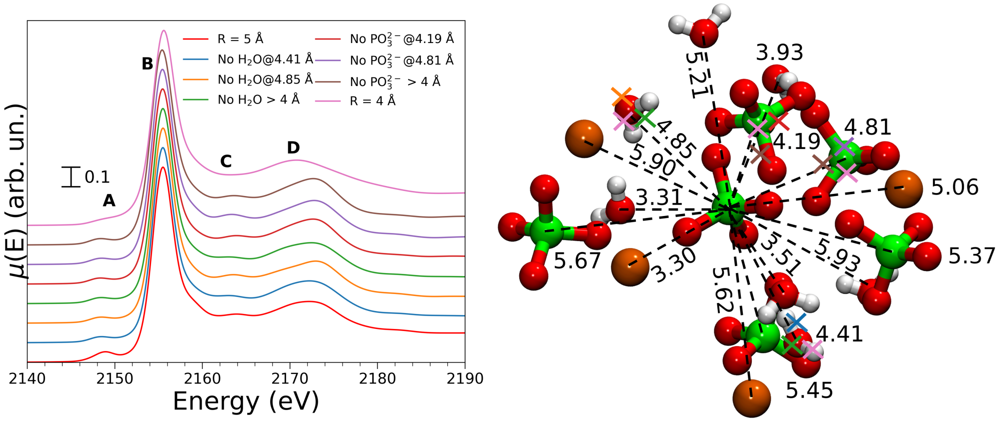

Figure S3: Left: comparison between the theoretical P K-edge XANES spectra of $FePO_4 \cdot 2H_2O$ (orthorombic phase) calculated at a cutoff of 4-5 Å and the spectra calculated by removing different H_2O or PO_4^{2-} species from the 5 Å cluster. Right: depiction of the 6 Å cluster where the species removed in the theoretical spectra shown in the left panel are indicated by color-coded cross marks. Color code for the elements: phosphorus, green, iron, brown, and oxygen, red.

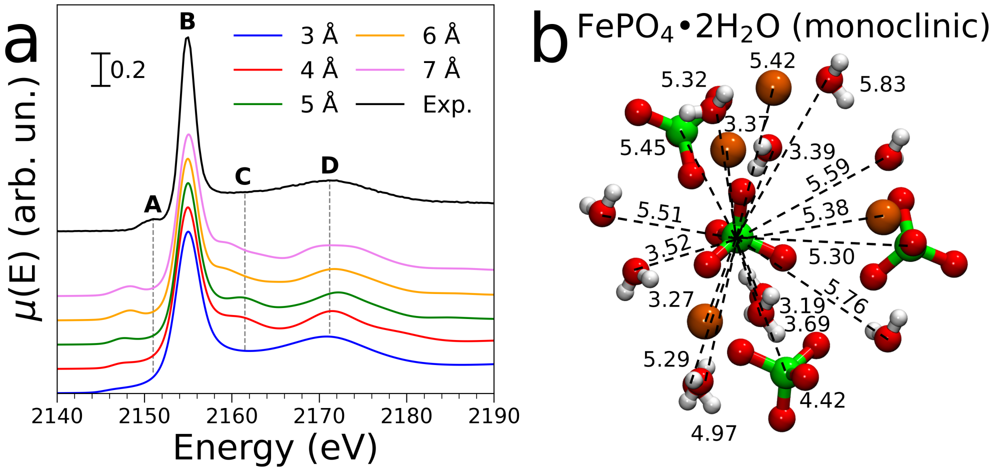

Figure S4: (a) Experimental P K-edge spectrum of $FePO_4 \cdot 2H_2O$ (monoclinic phase, black solid lines) compared to the theoretical spectra calculated using an increasing cutoff radius. Grey dashed lines highlight the energy position of the observed features. (d) Depiction of the structure employed in the calculation of the $FePO_4 \cdot 2H_2O$ (monoclinic phase) theoretical spectrum where distance-equivalent ions have been omitted for clarity. Distances from the photoabsorber to the central atom of each ion or molecule (Å) are reported above the dashed lines. Color code: phosphorus, green, iron, brown, oxygen, red, and hydrogen, white.

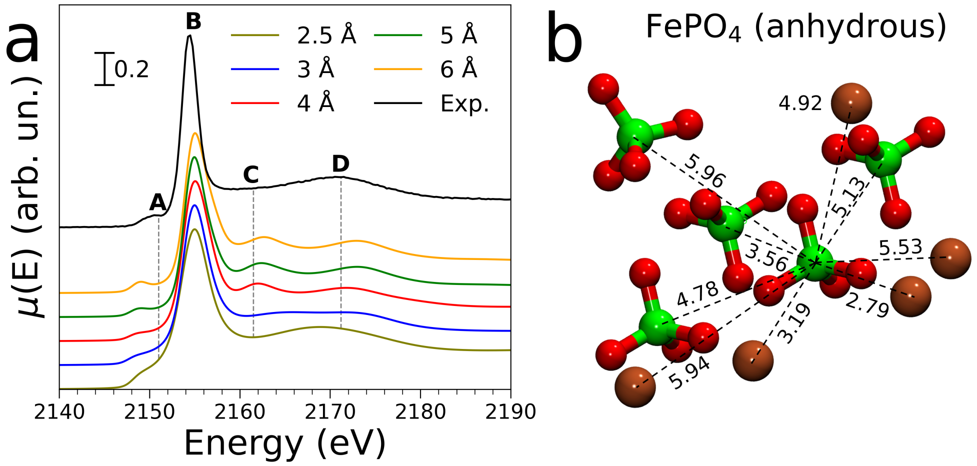

Figure S5: (a) Experimental P K-edge spectrum of $FePO_4$ (anhydrous, black solid lines) compared to the theoretical spectra calculated using an increasing cutoff radius. Grey dashed lines highlight the energy position of the observed features. (d) Depiction of the structure employed in the calculation of the $FePO_4$ (anhydrous) theoretical spectrum where distance-equivalent ions have been omitted for clarity. Distances from the photoabsorber to the central atom of each ion or molecule (Å) are reported above the dashed lines. Color code: phosphorus, green, iron, brown, and oxygen, red.

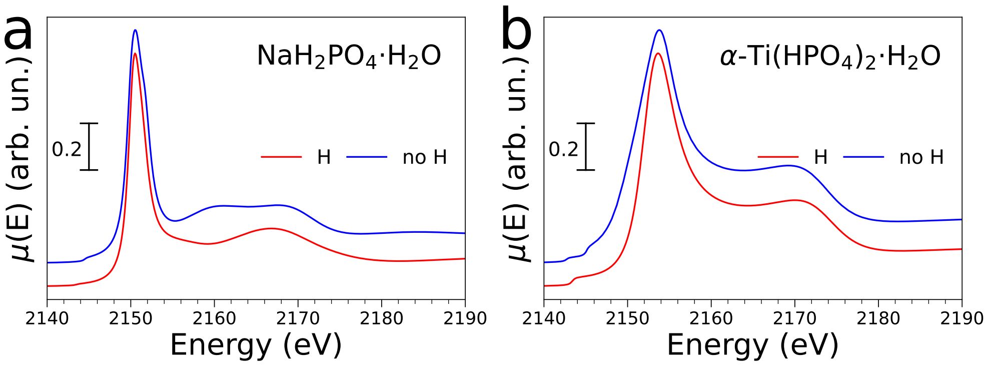

Figure S6: (a) Theoretical P K-edge XANES spectrum of the dihydrogen phosphate ion in $NaH_2PO_4{\cdot}H_2O$ calculated with (red curve) and without (blue curve) considering hydrogen atoms in the structure. (b) Theoretical P K-edge XANES spectrum of the monohydrogen phosphate ion in α-$Ti(HPO_4)_2{\cdot}H_2O$ calculated with (red curve) and without (blue curve) considering hydrogen atoms in the structure.

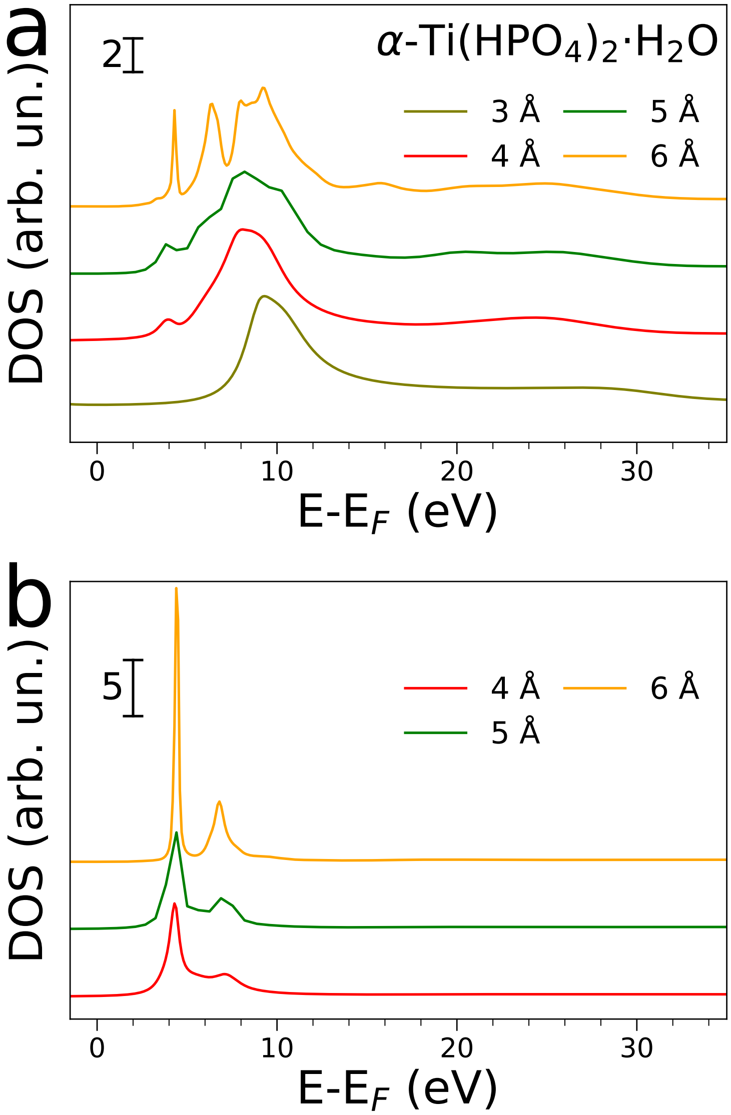

Figure S7: (a) P density of states (DOS) of α-Ti$(HPO_4)_2\cdot H_2O$ calculated using an increasing cutoff radius. (b) Ti density of states (DOS) of α-Ti$(HPO_4)_2\cdot H_2O$ calculated using an increasing cutoff radius.

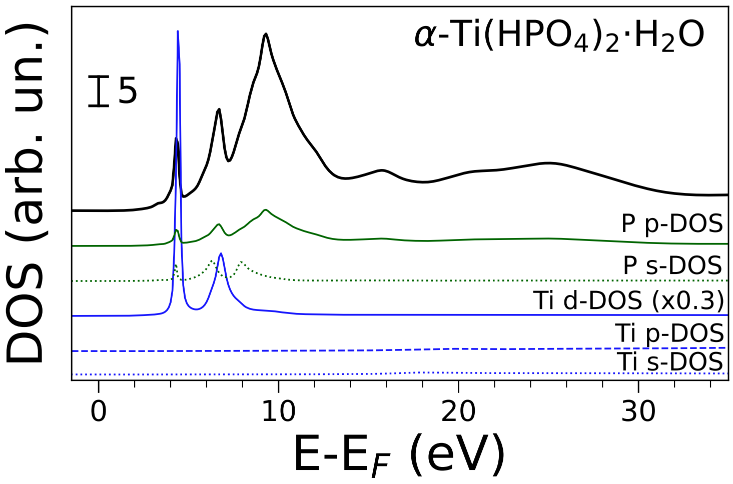

Figure S8: Theoretical spectrum of α-Ti(HPO_4)$_2$·H_2O (no convolution, black) and angular momentum projected density of states (DOS) of P (p, green solid line and s, green dotted line) and Ti (d, blue solid line, p, blue dashed line and s, blue dotted line) calculated using a cutoff radius of 6 Å. Both the theoretical spectrum and the Ti d-DOS have been scaled for better comparison.

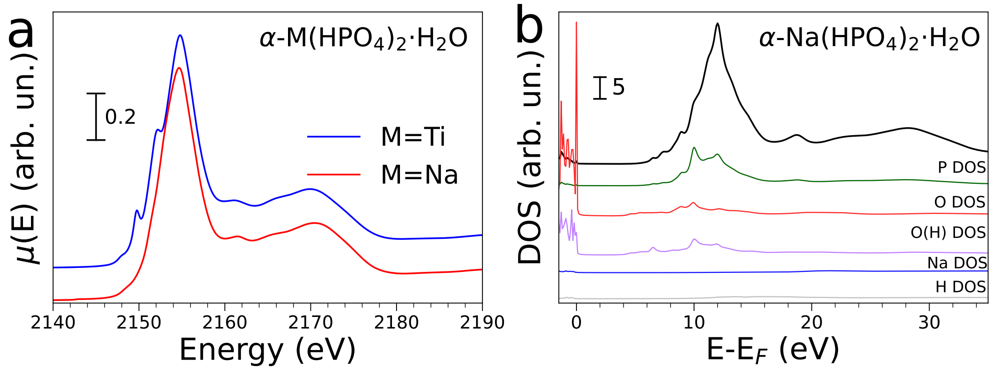

Figure S9: (a) Theoretical P K-edge XANES spectra of α-Ti(HPO_4)$_2$·H_2O (red solid line) and of an analogous structure obtained by replacing Ti with Na (blue solid line) calculated using a cutoff radius of 6 Å. (b) Theoretical P K-edge XANES spectrum of the latter structure (black, no convolution) compared to the density of states (DOS) of P (green), O (red for proton-free O, purple for OH), Na (blue), and H (grey) calculated using a cutoff radius of 6 Å. The theoretical spectrum has been scaled for better comparison.

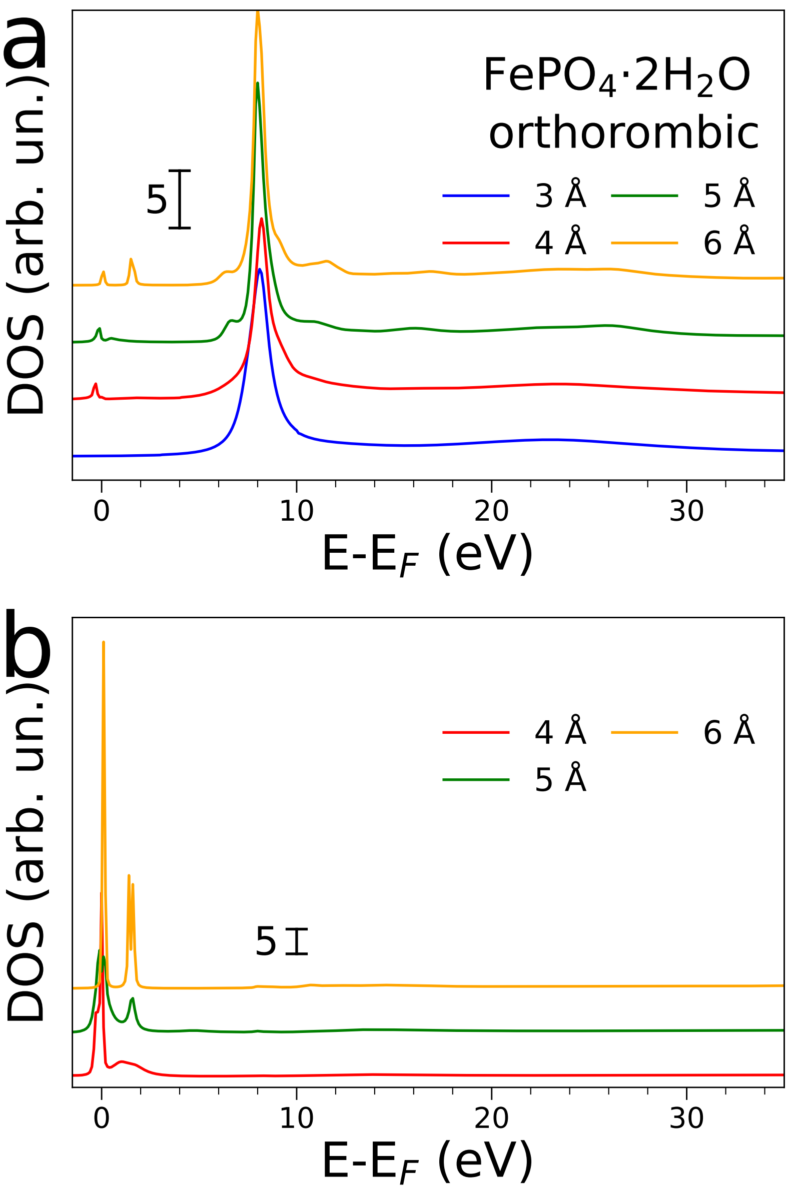

Figure S10: (a) P density of states (DOS) of $FePO_4{\cdot}2H_2O$ (orthorombic phase) calculated using an increasing cutoff radius. (b) Fe density of states (DOS) of $FePO_4$ calculated using an increasing cutoff radius.

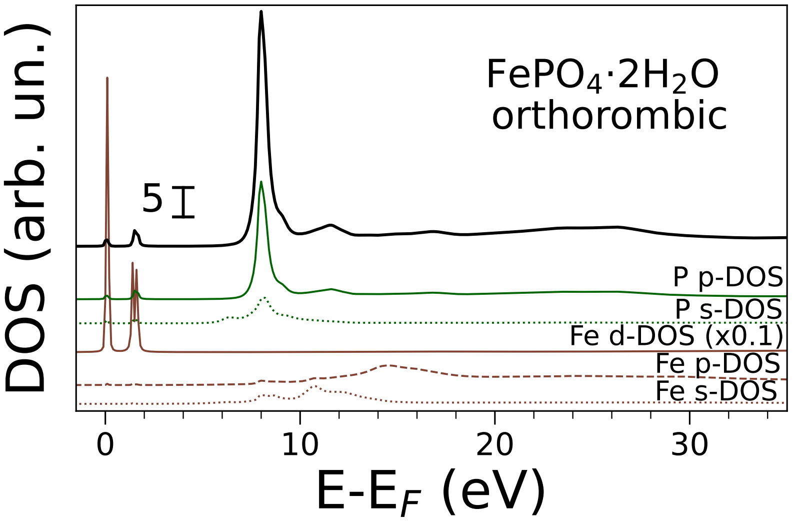

Figure S11: Theoretical spectrum of $FePO_4{\cdot}H_2O$ (orthorombic phase, no convolution, black) and angular momentum projected density of states (DOS) of P (p, green solid line and s, green dotted line) and Fe (d, brown solid line, p, brown dashed line and s, brown dotted line) calculated using a cutoff radius of 6 Å. Both the theoretical spectrum and the Fe d-DOS have been scaled for better comparison.

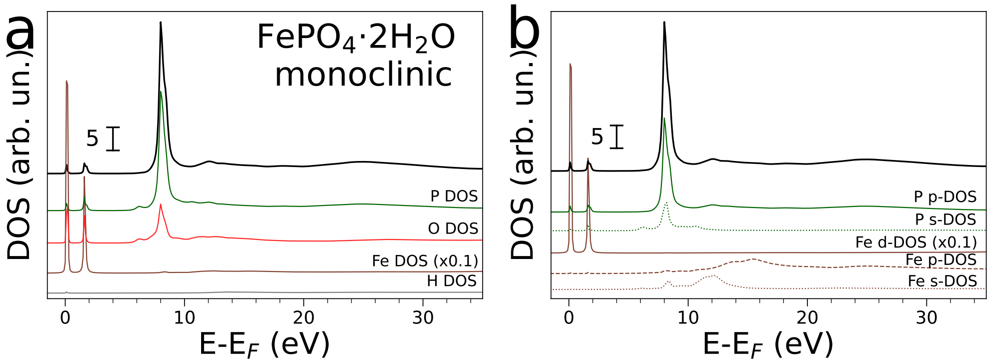

Figure S12: (a) Theoretical P K-edge XANES spectrum of $FePO_4{\cdot}2H_2O$ (monoclinic phase, black solid line, no convolution) and projected density of states (DOS) of of P (green), O (red for proton-free O, purple for OH), Fe (brown), and H (grey) calculated using a cutoff radius of 6 Å. (b) Theoretical P K-edge XANES spectrum of $FePO_4{\cdot}2H_2O$ (orthorombic phase, black solid line, no convolution) and angular momentum projected density of states (DOS) of P (p, green solid line and s, green dotted line) and Fe (p, brown solid line, and s, brown dotted line) calculated using a cutoff radius of 6 Å. The theoretical spectrum and Fe DOS have been scaled in both panels for better comparison.

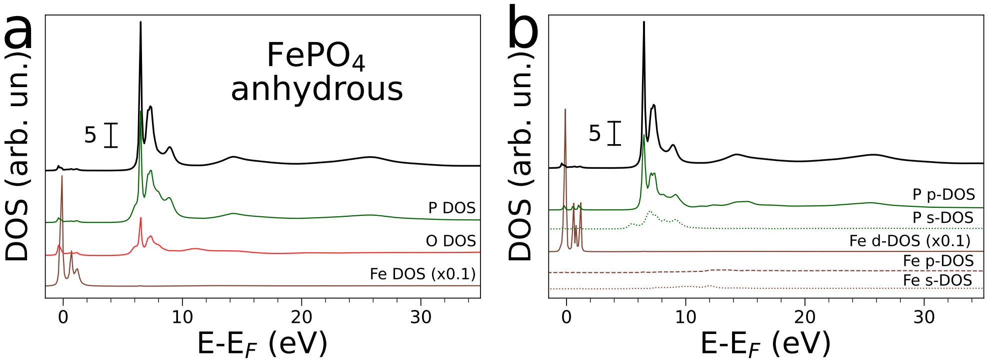

Figure S13: (a) Theoretical P K-edge XANES spectrum of $FePO_4$ (anhydrous, black solid line, no convolution) and projected density of states (DOS) of of P (green), O (red for proton-free O, purple for OH), Fe (brown), and H (grey) calculated using a cutoff radius of 6 Å. (b) Theoretical P K-edge XANES spectrum of $FePO_4$ (anhydrous, black solid line, no convolution) and angular momentum projected density of states (DOS) of P (p, green solid line and *s*, green dotted line) and Fe (p, brown solid line, and s, brown dotted line) calculated using a cutoff radius of 5 Å. The theoretical spectrum and Fe DOS have been scaled in both panels for better comparison.

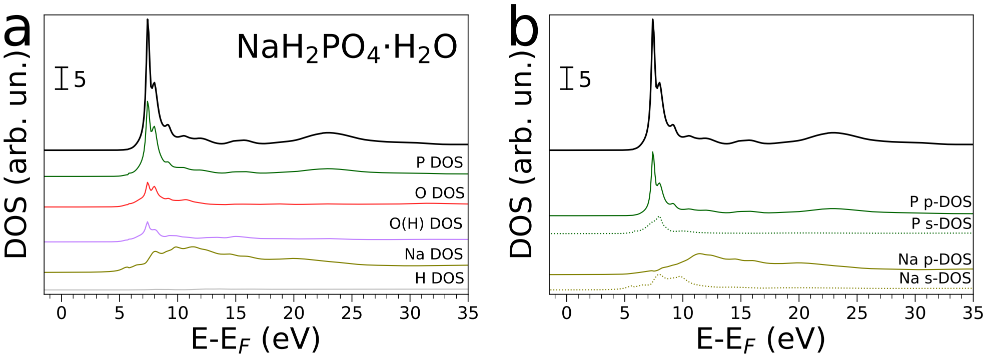

Figure S14: (a) Theoretical P K-edge XANES spectrum of $NaH_2PO_4{\cdot}H_2O$ (black solid line, no convolution) and projected density of states (DOS) of of P (green), O (red for proton-free O, purple for OH), Na (olive), and H (grey) calculated using a cutoff radius of 5 Å. (b) Theoretical spectrum of $NaH_2PO_4{\cdot}H_2O$ (no convolution, black) and angular momentum projected density of states (DOS) of P (p, green solid line and *s*, green dotted line) and Na (p, olive solid line, and s, olive dotted line) calculated using a cutoff radius of 5 Å. The theoretical spectrum has been scaled in both panels for better comparison.

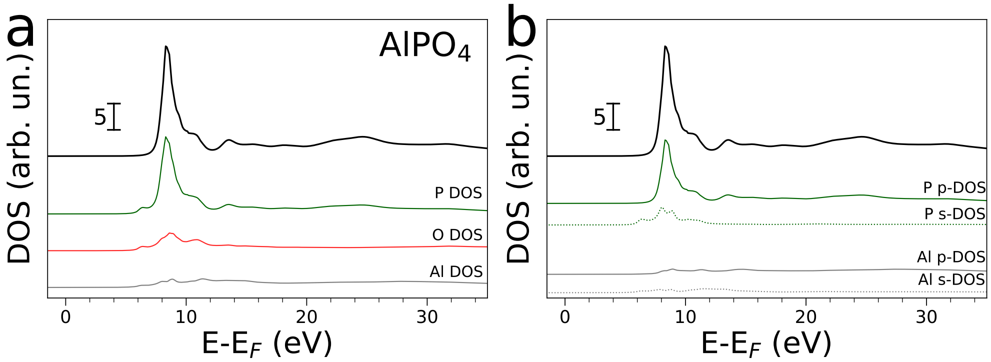

Figure S15: (a) Theoretical P K-edge XANES spectrum of $AlPO_4$ (black solid line, no convolution) and projected density of states (DOS) of of P (green), O (red for proton-free O, purple for OH), Na (olive), and H (grey) calculated using a cutoff radius of 6 Å. (b) Theoretical spectrum of $AlPO_4$ (no convolution, black) and angular momentum projected density of states (DOS) of P (p, green solid line and *s*, green dotted line) and Al (p, grey solid line, and s, grey dotted line) calculated using a cutoff radius of 6 Å. The theoretical spectrum has been scaled in both panels for better comparison.

3 Supplementary Tables (Table S1)

Table S1: Energy shifts applied to each theoretical spectrum.

$NaH_2PO_4 \cdot H_2O$	Cutoff radius (A)	3	4	5	6	
	Energy shift (eV)	2.0	1.3	1.5	1.4	
$AlPO_4$	Cutoff radius (A)	3	4	5	6	7
	Energy shift (eV)	1.2	0.2	0.7	0.7	0.8
α-$Ti(HPO_4)_2 \cdot H_2O$	Cutoff radius (A)	3	4	5	6	7
	Energy shift (eV)	-2.2	0.2	0.1	-0.4	-0.7
$FePO_4 \cdot 2H_2O$ (orthorombic)	Cutoff radius (A)	3	4	5	6	7
	Energy shift (eV)	-0.1	1.2	1.3	1.3	1.3
$FePO_4 \cdot 2H_2O$ (monoclinic)	Cutoff radius (A)	3	4	5	6	7
	Energy shift (eV)	-0.2	1.1	1.2	1.2	1.3
$FePO_4$	Cutoff radius (A)	3	4	5	6	
	Energy shift (eV)	2.6	2.1	2.3	2.4	2.5

References

(S1) Song, Y.; Zavalij, P. Y.; Suzuki, M.; Whittingham, M. S. New Iron(III) Phosphate Phases: Crystal Structure and Electrochemical and Magnetic Properties. *Inorg. Chem.* **2002**, *41*, 5778–5786.

(S2) Andersson, A. S.; Kalska, B.; Häggström, L.; Thomas, J. O. Lithium Extraction/Insertion in $LiFePO_4$: an X-ray Diffraction and Mössbauer Spectroscopy Study. *Solid State Ion.* **2000**, *130*, 41–52.
